# Quantification of the virus-host interaction in human T lymphotropic virus I infection

**DOI:** 10.1186/1742-4690-2-75

**Published:** 2005-12-09

**Authors:** Becca Asquith, Angelina J Mosley, Adrian Heaps, Yuetsu Tanaka, Graham P Taylor, Angela R McLean, Charles RM Bangham

**Affiliations:** 1 Department of Immunology, Imperial College, London W2 1PG, UK; 2 Department of Zoology, University of Oxford, Oxford OX1 3PS, UK; 3 Department of Immunology, Graduate School and Faculty of Medicine, University of the Ryukyus, Okinawa 903-0215, Japan; 4 Department of Genito-Urinary Medicine and Communicable Diseases, Imperial College, London W2 1PG, UK

## Abstract

**Background:**

HTLV-I causes the disabling inflammatory disease HAM/TSP: there is no vaccine, no satisfactory treatment and no means of assessing the risk of disease or prognosis in infected people. Like many immunopathological diseases with a viral etiology the outcome of infection is thought to depend on the virus-host immunology interaction. However the dynamic virus-host interaction is complex and current models of HAM/TSP pathogenesis are conflicting. The CD8+ cell response is thought to be a determinant of both HTLV-I proviral load and disease status but its effects can obscure other factors.

**Results:**

We show here that in the absence of CD8+ cells, CD4+ lymphocytes from HAM/TSP patients expressed HTLV-I protein significantly more readily than lymphocytes from asymptomatic carriers of similar proviral load (P = 0.017). A high rate of viral protein expression was significantly associated with a large increase in the prevalence of HAM/TSP (P = 0.031, 89% of cases correctly classified). Additionally, a high rate of Tax expression and a low CD8+ cell efficiency were independently significantly associated with a high proviral load (P = 0.005, P = 0.003 respectively).

**Conclusion:**

These results disentangle the complex relationship between immune surveillance, proviral load, inflammatory disease and viral protein expression and indicate that increased protein expression may play an important role in HAM/TSP pathogenesis. This has important implications for therapy since it suggests that interventions should aim to reduce Tax expression rather than proviral load *per se*.

## Background

Human T-Lymphotropic Virus Type I (HTLV-I) is a persistent retrovirus. The majority of infected individuals remain lifelong, asymptomatic carriers of the virus (ACs). However, 2–3% of infected individuals develop an aggressive malignancy named Adult T cell Leukemia. A further 2–3% develop inflammatory disease of one or more organs. The best characterised inflammatory disease is HTLV-I-associated myelopathy/ tropical spastic paraparesis (HAM/TSP), a chronic inflammatory condition of the central nervous system. It is not understood why most HTLV-I infected individuals remain asymptomatic yet some develop inflammatory disease.

The two factors most often associated with HAM/TSP are high proviral load [1] and high HTLV-I-specific CD8+ cytotoxic T lymphocyte (CTL) frequency [2,3], suggesting that virus-host immunology interactions are important in determining the outcome of infection. It is not known whether the HTLV-I-specific CD8+ cellular response is pathogenic and contributes to the tissue damage in HAM/TSP, or whether it is protective and reduces proviral load and the risk of the development of HAM/TSP. There is evidence supporting both pictures [3-8], and they are not necessarily mutually exclusive [9]. What is clear is that a good understanding of the CTL-virus interaction is crucial to understanding the control of HTLV-I infection and the progression to HAM/TSP.

Although a high proviral load is associated with HAM/TSP there is large amount of overlap in proviral load between HAM/TSP patients and ACs [1]. There exist ACs with high proviral loads (> 3% PBMC infected) and HAM/TSP patients with low proviral loads (<1% PBMC infected), indicating that a high proviral load is neither necessary nor sufficient to cause HAM/TSP. Current theories of HAM/TSP pathogenesis postulate excess activation of CD4+ and/or CD8+ lymphocytes [5,6,10]. We reasoned that this was more likely to be directly associated with the amount of viral antigen rather than the amount of proviral DNA. However, investigation of viral antigen is confounded by the presence of CD8+ cells which effectively kill HTLV-I-expressing cells *ex vivo *[11-13], and presumably *in vivo *[3,7]. We therefore investigated viral protein expression in cells from HAM/TSP patients and ACs following *ex vivo *CD8+ cell depletion with the aim of quantifying the relative importance of proviral load, viral protein expression and CTL surveillance in HTLV-I infection.

## Results

### Tax expression was higher in HAM/TSP patients than ACs

**Figure 1:**
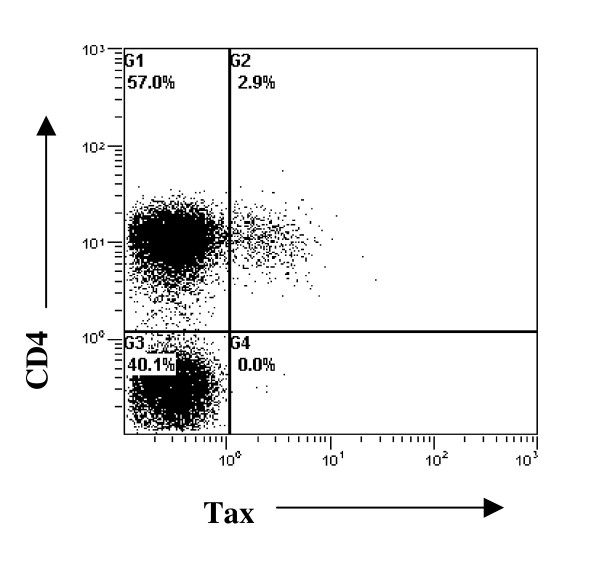
**Representative Tax staining**. Tax expression in CD4+ cells was measured by flow cytometry. Tax and CD4 co-staining from a representative subject is shown.

**Figure 2:**
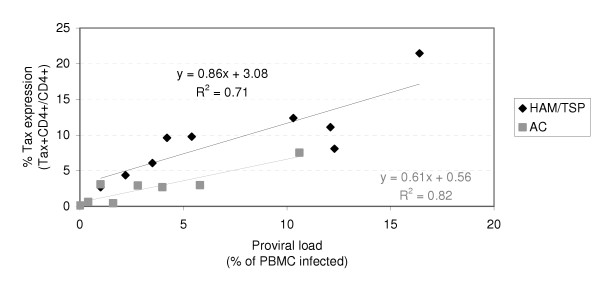
**Tax expression in CD4+ lymphocytes from HTLV-I infected individuals**. The proportion of CD4+ cells expressing the viral protein Tax after 18 h *ex vivo *incubation in the absence of CD8+ cells was measured by flow cytometry. Tax expression was significantly higher in lymphocytes from HAM/TSP patients than from ACs of comparable proviral load (ANOVA, two tailed test p = 0.014. Permutation test two-tailed test p = 0.017). This result was robust to removal of outliers: the P value either remained unchanged or decreased on removal of outliers.

Tax protein is the first HTLV-1 protein to be expressed in an infected cell; we therefore focused on Tax protein as an index of HTLV-1 proviral expression. Tax protein expression is usually below the detection limit in lymphocytes immediately *ex vivo *but increases spontaneously over time during culture [12]; we therefore measured the proportion of CD4+ lymphocytes expressing Tax after 18 h. CD8+ cells were depleted prior to culture to prevent lysis of Tax-expressing cells. Tax expression was measured in 16 patients; representative staining is shown in Fig. 1, results from all subjects are shown in Fig. 2.

Tax expression (i.e. the proportion of CD4+ cells expressing Tax protein) at any given proviral load was significantly higher in the HAM/TSP patients than in the ACs (permutation test P = 0.017, ANOVA P = 0.014 two tailed). Grouping the patients by proviral load (Table I) showed that Tax expression was 2.5–3 times higher in HAM/TSP patients compared with ACs of similar proviral loads. We found that the median probability of an infected cell expressing Tax after 18 h *ex vivo *culture was 50% in HAM/TSP patients and 28% in ACs (Methods).

**Table 1:** Tax expression in CD4^+ ^lymphocytes is 2.5–3 fold higher in HAM/TSP patients than ACs of comparable proviral load

		Patient	Proviral load (% PBMC)	Rate of CTL lysis (per CD8+ cell per day)	%Tax expression (Tax+CD4+/CD4+)	Mean Tax expression	Fold Increase in Tax Expression (HAM÷AC)
Group 1	AC	HBD	0.0	0.220	0.1	1.3	2.8
		HT	1.0	0.062	3.1		
		HY	0.4	0.065	0.6		

	HAM	TAQ	1.0	0.083	2.7	3.5	
		TAY	2.2	0.298	4.4		

Group 2	AC	HBH	4.0	0.020	2.7	2.8	3.0
		HBF	2.8	0.029	2.9		

	HAM	TAT	4.2	0.058	9.6	8.5	
		TAU	5.4	0.049	9.8		
		TBA	3.5	0.050	6.1		

Group 3	AC	HS	5.8	0.001	3.0	5.2	2.5
		HAY	10.6	-0.007	7.5		

	HAM	TW	10.3	0.024	12.4	13.3	
		TAC	12.1	0.091	11.1		
		TBG	16.4	0.007	21.5		
		TBI	12.3	0.003	8.1		

The choice of groups of "comparable" proviral load is, to some extent, subjective but a range of alternative groupings gave similar results. This included a grouping in which the mean proviral load of ACs was higher than the mean proviral load of HAM/TSP patients in each group (it was necessary to omit some high proviral load HAM/TSP patients in order to obtain this alternative grouping). We have illustrated our results using this particular grouping because it is a representative grouping and because it yields two or more subjects in each group thus minimising the effect of outliers.

### Tax expression and risk of HAM/TSP

We analysed the association between proviral load, Tax expression and clinical status using logistic regression. In our patient sample, although there was a trend for a higher proviral load in HAM/TSP patients, there was no significant association between proviral load and HAM/TSP. In contrast we found that Tax expression was a significant predictor of disease. This was true whether we considered the proportion of CD4+ lymphocytes that were Tax+ (fraction of cases correctly classified = 82%; P = 0.031) or the rate of Tax expression (proportion of Tax+ lymphocytes at a given proviral load) (fraction of cases correctly classified = 89%; P = 0.035). The odds of having HAM/TSP were 20 fold higher in the subjects with a high rate of Tax expression compared to the subjects with a low rate of Tax expression (P = 0.02). A high rate of Tax expression is therefore significantly associated with the disease HAM/TSP, independently of proviral load.

### Tax expression and the control of proviral load

Next we identified factors significantly associated with a high proviral load, using multiple regression. The factors considered were Tax expression and CTL lysis rate of infected cells *ex vivo*. The rate at which an individual's CTLs killed infected cells was measured during an 18 h *ex vivo *"CD8+ cell mediated anti-viral efficacy" assay (Methods, Table I). Proviral load and the proportion of CD4+ cells that were Tax+ were strongly positively correlated as expected. If the proportion of CD4+ cells that were Tax+ is used as a predictor variable then this will result in a highly significant model with a large proportion of the between-individual variation in proviral load "explained". However, this will simply be because we have identified a surrogate marker for proviral load. Instead, we consider Tax expression after correcting for proviral load. That is, we divide the patient sample into those whose infected cells have a high probability or rate of Tax expression (high proportion of Tax+CD4+ cells at a given proviral load after 18 h culture) and those whose infected cells have a low rate of Tax expression. We then asked whether subjects with high and low rates of Tax expression with equally efficacious CTL responses (similar rates of CTL lysis of infected cells) had different proviral loads. It was found that the rate of Tax expression (high or low) was a significant predictor of proviral load (P = 0.005, 13% of proviral load predicted) independent of the CTL lysis rate, which was also a significant predictor (P = 0.003, 30% of proviral load predicted). Overall, 43% of the between-individual variation in proviral load could be explained by variation in these two parameters. We conclude that the rate of CTL-mediated lysis and the rate of Tax expression are significant independent predictors of HTLV-I proviral load.

### Why is an increased rate of Tax expression associated with an increased proviral load?

**Figure 3:**
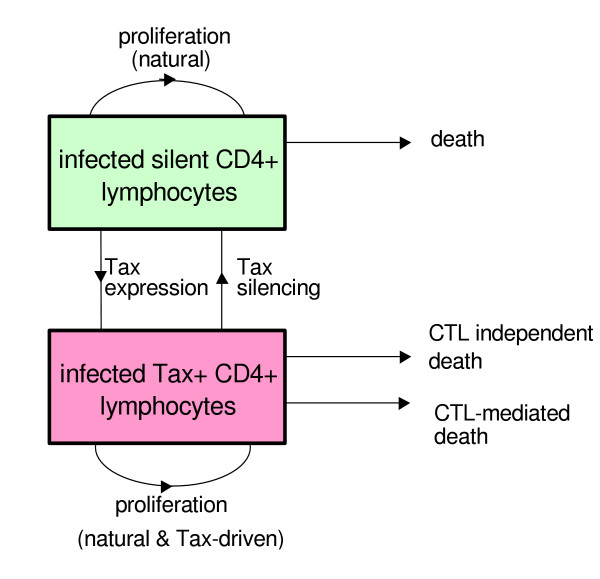
**Schematic of the general model to describe the relationship between Tax+ and Tax- infected cells *in vivo***. Death of silently infected CD4+ cells will include all normal cell death processes such as necrosis and apoptosis. Death of Tax-expressing CD4+ cells is divided into two: that which can be directly attributed to CTL (e.g. perforin mediated lysis or Fas-mediated apoptosis) and that which is independent of CTL (including normal cell necrosis and apoptosis as well as Tax-induced apoptosis and activation induced cell death). Natural proliferation describes the normal background rate of CD4+ cell turnover. Tax-driven proliferation describes the extra proliferation that may be caused by Tax expression due to its upregulation of cellular genes involved in cell proliferation and deregulation of cell cycle checkpoints [15–17, 33].

**Figure 4:**
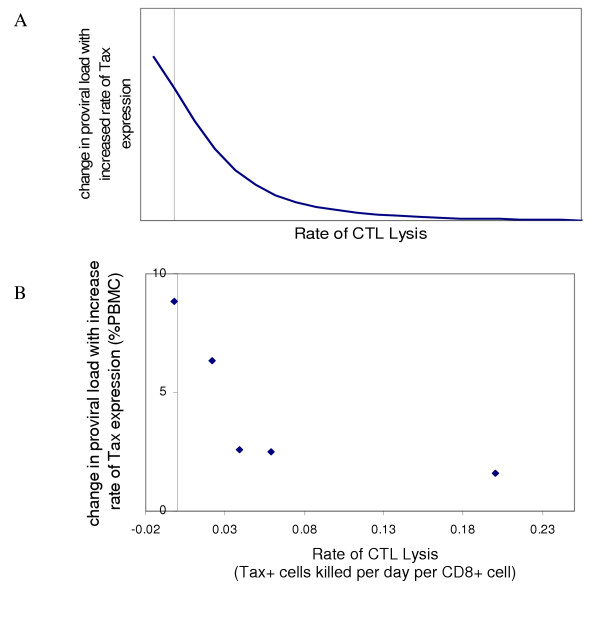
**The increase in proviral load due to a high rate of Tax expression decreases with increasing rate of CTL lysis of infected cells**. A theoretical model suggests that one explanation for the increase in proviral load associated with a high rate of Tax expression is that expression of Tax promotes cell division. The model predicts (4A) that the difference in proviral load between individuals who have high and low rates of Tax expression decreases as the CTL lysis rate increases. The experimental data (4B) are consistent with this prediction. The experimental data "change in proviral load with increased rate of Tax expression" was calculated by grouping all 16 subjects into groups of similar rates of lysis. Within each group the mean proviral load of the subjects with a high rate of Tax expression and the mean proviral load of subjects with a low rate of Tax expression was calculated. The difference between these two means is the "change in proviral load with an increased rate of Tax expression" and was plotted against the average rate of CTL lysis in that group.

Initially it would seem that a high rate of Tax expression should be associated with a low proviral load since it would result in the exposure of a high proportion of infected cells to the immune response (as well as possibly having a toxic or pro-apoptotic effect [14]). We therefore modelled this to understand how an increased rate of Tax expression could lead to increased proviral load at a given CTL lysis rate. The model is represented in diagrammatic form in Fig. 3 (details in Additional file 1). The model predicted that Tax expression could increase proviral load because, although Tax expression exposes infected cells to the CTL response, it can also increase infected cell proliferation by upregulating cellular genes involved in proliferation and deregulating cell cycle checkpoints [15-17]. The balance between CTL killing and Tax-driven mitosis determines the net effect of increased Tax expression on proviral load. If the CTL response is weak then the increase in proviral load due to a high rate of Tax expression is large. If the CTL response is stronger then the increase in proviral load conferred by a high rate of Tax expression decreases. That is, the model predicted that the gain in proviral load conferred by a high rate of Tax expression should fall as the CTL lysis rate increases (Fig. 4A). To test if the experimental data fulfils this prediction we grouped the 16 subjects into groups of similar CTL lysis rate, then subtracted the mean proviral load of subjects with a low rate of Tax expression from the mean proviral load of subjects with a high rate of Tax expression within each group. On plotting this difference against the mean CTL lysis rate for the group (Fig. 4B) it can be seen that the experimental data accord with the prediction, with a progressive decrease in difference between the proviral load of subjects with high and low rates of Tax expression as the lysis rate of the CTL response increases.

## Discussion

Two earlier studies have quantified Tax mRNA in HTLV-I infection [18,19]. These papers reported conflicting results. Furukawa *et al*. [18] reported that there was no difference in Tax mRNA levels between HAM/TSP patients and ACs after variation in proviral load had been accounted for. In contrast, Yamano *et al*. [19] reported that Tax mRNA remained significantly higher in HAM/TSP patients after correction for proviral load. Our work extends this earlier research by investigating Tax protein rather than mRNA and, most importantly, by removing the potentially confounding factor of the CD8+ cell response. Earlier work was done in the presence of CD8+ cells, making it hard to interpret since it was not known how much of the between-individual variation in Tax mRNA was attributable to variation in the HTLV-I-specific CTL response. In particular, systematic differences in the frequency of HTLV-I specific CTLs between ACs and HAM/TSP patients are widely reported [2,5]; it was possible that this difference was sufficient to explain the reported differences in Tax mRNA. Measuring Tax protein expression by flow cytometry also provides information at a per cell level enabling us to determine whether an increase in Tax expression is due to an increase in the number of cells expressing Tax or an increase in the amount of Tax expressed per Tax expressing cell -a refinement that is not possible with RT-PCR.

We found that Tax expression (proportion of CD4+ cells expressing Tax) was a significant predictor of HAM/TSP status in our patient sample. Interestingly, this was in a subject group where proviral load was not significantly associated with HAM/TSP. We can therefore be confident that the association between Tax expression and HAM/TSP is not simply because Tax expression acted as a surrogate for proviral load *per se*. On the other hand, the association between proviral load and HAM/TSP reported in the Japanese population [1] may not result from a high proviral load increasing the risk of disease as is often assumed. Instead, it could be that high Tax expression causes HAM/TSP and that since Tax expression and proviral load are correlated this is manifest as an association between proviral load and HAM/TSP. Current hypotheses of HAM/TSP pathogenesis centre around excess activation of CD4+ and/or CD8+ lymphocytes [6,20,21]. That excess T cell activation should be associated with the expression rather than simply the possession of a provirus is intuitively reasonable. HAM/TSP pathogenesis remains poorly understood and surprisingly few factors have been identified that distinguish HAM/TSP patients from ACs. The observation that high Tax expression is significantly associated with HAM/TSP, the odds of having HAM/TSP being 20 fold higher in subjects with a high rate of Tax expression compared with subjects with a low rate of Tax expression, is an important step towards identifying why some individuals develop HAM/TSP but most remain asymptomatic. The absence of a significant association between proviral load and HAM/TSP in our subject group could be because the overlap in proviral load between HAM/TSP patients and ACs, which is considerable in the Japanese population [1], is even broader in our, mainly Afro-Caribbean population.

We also report that subjects with a high rate of Tax expression have high proviral loads. We suggest that although Tax expression exposes infected cells to the CTL response it also increases infected cell proliferation by upregulating cellular genes involved in proliferation and deregulating cell cycle checkpoints. This increase in infected cell proliferation results in a net increase in proviral load at a given CTL strength. If the CTL response is weak then the "benefit" to the virus of a high rate of Tax expression is very large, resulting in a considerably higher proviral load than a low rate of Tax expression. If the CTL response is stronger then the "benefit" conferred by a high rate of Tax expression decreases. If the CTL strength is extremely high then the virus "benefits" from remaining silent. Consistent with this explanation we found that the increase in proviral load associated with a high rate of Tax expression was reduced in subjects with a strong CTL response (high rate of lysis of infected cells). It might be expected that this would drive within-host evolution of HTLV-I with low Tax-expressing strains being selected for in individuals with a strong CTL response and vice versa. However, HTLV-I has, compared to other retroviruses, little scope for within-host evolution due to the low frequency of variant strains [22]. So, although virus infecting an individual with a very strong immune response may benefit from reduced Tax expression this will not necessarily result in the emergence of new virus variants. Why Tax expression should vary between individuals is not known and is the subject of ongoing research. Possible reasons include differences between individuals in the proportion of defective proviruses, in CD8+ cell-independent immunity, in proviral integration site or in epigenetic alterations to the proviral DNA such as methylation. Finally, Tax expression may be affected by the expression of other HTLV-1 regulatory proteins such as p30, HBZ and Rex [23-25]. It is possible that increased Tax expression could explain the reported associations between HAM/TSP and HTLV-I phylogenetic subgroup [26] since variations in the viral LTR could result in increased rates of Tax expression.

The relationship between Tax expression *ex vivo *and Tax expression *in vivo *is not fully understood. The fact that Tax expression *ex vivo *is significantly associated with disease status suggests that Tax expression *ex vivo *and Tax expression *in vivo *are correlated. More direct evidence of this correlation is provided by a recent study of *in vivo *CD4+ T lymphocyte kinetics in HTLV-I infected subjects in which it was found that cells that expressed Tax *ex vivo *had proliferated more rapidly *in vivo *than cells from the same individual that did not express Tax [27].

## Conclusion

In summary, we present two main findings. We have quantified the contribution of viral protein expression and CTL lysis of infected cells to proviral load, finding that a low CTL lysis rate and a high rate of Tax expression are independently significantly associated with a high proviral load (P = 0.003, P = 0.005 respectively) and suggested causal mechanisms for both of these relationships. Importantly, we also find that a high rate of Tax expression is a significant risk factor associated with HAM/TSP (P = 0.017) and that the rate of Tax expression correctly classifies 89% of infected subjects. We propose that high Tax expression rather than high proviral load is causally associated with HAM/TSP pathogenesis. If correct, this conclusion implies that therapeutic intervention should aim to reduce Tax expression rather than proviral load *per se*.

## Methods

### Subjects

All subjects attended the HTLV-I clinic at St Mary's Hospital, London and gave informed consent. The study was approved by the Local Research Ethics Committee of St Mary's Hospital NHS Trust and all procedures were carried out in accordance with the Declaration of Helsinki. HTLV-I infection was diagnosed by the presence of antibodies to HTLV-I Gag and Env antigens in sera by Western blot and confirmed by detection of HTLV-I Tax by DNA PCR. Diagnosis of HAM/TSP was made following World Health Organisation criteria. 16 HTLV-I infected subjects were studied, the median age was 60 yrs (range 36–74 yrs). One subject (HS) was studied on two separate occasions (6 mths apart); as both proviral load and Tax expression had changed both data points were included in our analysis. Exclusion of one or the other of the data points did not qualitatively alter any of the results.

### Measurement of Tax expression

CD8^+ ^cells were positively selected from thawed cryopreserved PBMC using magnetic microbeads (Miltenyi Biotec). The CD8- fraction was washed twice and resuspended in standard culture medium (total volume 1 ml) in 5 ml round-bottomed, vented capped tubes. After 18 hours' culture at 37°C, 5% CO2, the cells were washed in PBS, fixed for 20 mins at room temperature in 2% paraformaldehyde (pH 7.4; Sigma), washed then surface stained for CD4 and CD8 antigens by incubation at room temperature for 20 mins in PBS/7% Normal Goat Serum with relevant mAbs (15 μg/ml of PC5-conjugated anti-CD4 and ECD-conjugated anti-CD8; Beckman Coulter). The cells were washed once and stained intracellularly for Tax protein [12] using the Tax monoclonal antibody Lt-4 [28], then analysed by flow cytometry on a Coulter EPICS XL. All assays were done in duplicate and the proportion of CD4+ lymphocytes that were Tax positive was calculated. The average purity of CD8- cells was 96%, minimal purity was 88%.

### Proviral load measurement

Proviral load was measured as previously described [13]. Briefly DNA from PBMC was amplified for HTLV-I DNA (Tax specific primers as in [29,30]) and β-actin by real time quantitative PCR. Standard curves were generated using DNA from the C10 cell line. The sample copy number was estimated by interpolation from the standard curve, calculated as an average of three dilutions and expressed as the proportion of HTLV-I infected PBMC, assuming one provirus per infected cell [31].

### Measurement of CD8+ cell lysis of Tax-expressing cells

CD8+ cell lysis was measured using an *ex vivo *"CD8+ cell mediated anti-viral efficacy" assay as previously described [13]. Briefly, CD8+ and CD8- cell fractions were isolated from PBMC using magnetic microbeads; washed, resuspended in standard culture medium and aliquotted into 5 ml round-bottomed, vented capped tubes at 3 to 6 different CD8+:CD8- ratios (lower, including and higher than the subject's normal ratio). No mitogens, cytokines or artificial peptides were added. After 18 hours' culture at 37°C, 5% CO_2_, the cells were washed in PBS, fixed for 20 mins at room temperature in 2% paraformaldehyde (pH 7.4; Sigma), washed then surface stained for CD4 and CD8 antigens (as described above). The cells were washed once and stained intracellularly for Tax (as described above), then analysed by flow cytometry on a Coulter EPICS XL. 30,000 events were routinely collected. All assays were done in duplicate.

The resulting data (the proportion of Tax+CD4+ cells surviving at different CD8+:CD8- ratios) was analysed mathematically. The CD8+ cell lysis rate, i.e. the rate at which Tax+CD4+ cells were killed by CD8+ cells, was estimated in each subject using the following model:



where *y *is the proportion of CD4+ cells expressing Tax (i.e. Tax+ CD4+ cells/CD4+ cells), *c *is the rate of increase of Tax expression, ε is the CD8^+ ^cell mediated lysis rate and *z *is the proportion of lymphocytes that are CD8+. This model was solved analytically and fitted to the data using nonlinear least squares regression, providing an estimate of the lysis rate (ε) in each individual. We have previously shown [13] that the CD8+ cell-mediated loss of Tax expressing cells was due to cell death (by propidium iodide staining); was perforin-dependent (i.e. is blocked by the perforin inhibitor concanamycin A) and was MHC class I restricted.

### Permutation test: Tax expression at a given proviral load

A permutation test [32] was used to test the null hypothesis "the proportion of CD4+ cells expressing Tax at a given proviral load is the same in HAM/TSP patients and ACs" in a model independent way (ANOVA assumes a linear relationship between Tax expression and proviral load). This was done by grouping the data into bins of similar proviral load. The binning algorithm used was to start from the lowest proviral load and then extend the boundary of the bin until at least one HAM/TSP and one AC data point were included. A boundary was then drawn and the next bin started. The maximum number of bins that could be constructed was 6. The mean frequency of Tax expressing cells (Tax+CD4+/CD4+) in the HAM/TSP patients and in the ACs in each of the 6 bins was calculated. The test statistic, "number of bins in which the proportion of CD4+ cells expressing Tax was higher in the HAM/TSP patients than the ACs" was counted.

The distribution of the test statistic under the null hypothesis was estimated using a Monte Carlo approach. That is, the AC and HAM/TSP labels were removed from the proviral load-Tax expression data pairs and randomly reassigned. The resulting "data" was binned using the algorithm defined above, and the test statistic calculated. This was repeated 1,000 times to estimate the distribution of the test statistic. The distribution was estimated in 10 different runs to check that it was stable. Using the resulting distribution, the probability of observing the test statistic under the null hypothesis was estimated and doubled to obtain a two-tailed P value. The grouping of subjects produced by the algorithm was Bin 1 TAQ, HT, HY, HBD; Bin 2 TAY, HSa; Bin 3 TBA, HBF; Bin 4 TAT, HBH; Bin 5 TAU, HSb; Bin 6 TW, TAC, TBI, TBG, HAY.

### Definition: high/ low rate of Tax expression

The sample group was divided into subjects whose provirus-positive cells had a high or low rate of Tax expression i.e. into subjects with a high or low proportion of Tax+CD4+ cells at a given proviral load after 18 h culture. This was done by fitting a straight line through the pooled HAM/TSP and AC proviral load-Tax expression data using linear regression. Subjects lying above this line were classed as having a high rate of Tax expression (high frequency of Tax+ cells at a give proviral load), subjects lying below it were classed as having a low rate of Tax expression (low frequency of Tax+ cells at a given proviral load). The figure in Additional file 2 illustrates this classification. Duplicate measurements of the frequency of Tax+ cells were made. For every subject except TAC both duplicates yielded the same classification into a high or low rate of Tax expression. We therefore excluded TAC from any analysis requiring this classification but always checked that including TAC as having either a low or a high rate of Tax expression did not qualitatively alter the results. We use "rate" of Tax expression to refer to the rate at which silently infected (i.e. provirus positive, viral protein negative) cells express Tax. This enables us to distinguish between the absolute level of Tax expression and the rate (or probability) of a silently infected cell expressing Tax.

### Logistic regression: predictors of disease status

Logistic regression was used to quantify the contribution of Tax expression to the odds of having HAM/TSP in our patient sample. Tax expression was considered in two ways: 1) as a continuous variable: % of CD4+ cells that are Tax+ after 18 h *ex vivo *culture and 2) as a dichotomous variable: high/low rate of Tax expression (frequency of Tax+CD4+ cells at a given proviral load) as defined above.

### Multiple regression: predictors of proviral load

Multiple linear regression was used to identify predictors of proviral load across all individuals. Three independent variables were considered: CTL lysis rate (continuous), rate of Tax expression (dichotomous: high/low) and a constant. Models were constructed by forwards and backwards stepwise procedures. The optimal model was

Ln [*pvl*] = -*A*(CTL lysic rate) + *b*(if rate of Tax expression = high).

Using this model the fraction of the observed variation in proviral load that could be explained by the variation in CTL lysis rate and rate of Tax expression was calculated. The significance of predictors quoted is the significance of that variable given the other variable in the regression equation.

### Probability of an infected cell expressing Tax in 18 h

To estimate the probability of an infected (provirus-positive) cell expressing Tax in 18 h we expressed the fraction of CD4+ cells that were Tax+ after 18 h culture as the fraction of infected cells that were Tax+ after 18 h using the formula



In this calculation we made the simplifying assumption that all proviral load was carried in CD4+ cells.

### Grouped data: relationship between Tax expression, proviral load and CTL lysis rate

A theoretical model (Fig. 3) predicted that the difference in proviral load between subjects with a high and low rate of Tax expression would decrease as the CTL lysis rate increased (Fig. 4A). To test this prediction the subjects were grouped into "bins" of similar lysis rate. The binning algorithm used was to start from the lowest CTL lysis rate and then extend the boundary of the bin until at least one subject with a high rate of Tax expression and one subject with a low rate of Tax expression were included (using the definition of rate of Tax expression given above). At that point a boundary was drawn and the next bin started. The maximum number of bins that could be obtained was 5. The difference in mean proviral load between the subjects with a high rate of Tax expression and the subjects with a low rate of Tax expression in each of the 5 bins was calculated and plotted against the mean CTL lysis rate in that bin (Fig. 4B). The grouping of subjects produced by the algorithm was Bin 1 TBG, TBI, HSa, HAY, HSb; Bin 2 TW, HBH; Bin 3 TAU, HBF; Bin 4 TAT, TBA, HT, HY; Bin 5 TAY, TAQ, HBD, TAC.

## Abbreviations

AC: asymptomatic carrier, CTL: cytotoxic T lymphocyte, HAM/TSP: HTLV-I associated myelopathy/ tropical spastic paraparesis, HTLV-I: Human T Lymphotropic Virus-I.

## Competing interests

The author(s) declare that they have no competing interests.

## Authors' contributions

BA conceived of and designed the study, performed the analysis and wrote the manuscript. AJM performed the Tax staining. AH & ARM contributed to the data interpretation. YT provided reagents. GPT recruited and monitored the subjects. CRMB helped design the study and draft the manuscript and contributed to data interpretation.

## Supplementary Material

Additional File 1 Description of the general model to describe the relationship between Tax+ and Tax- infected cells *in vivo*. Click here for file

Additional File 2 Figure illustrating the classification of the subject group into individuals whose provirus-positive cells had a high or low rate of Tax expression. Click here for file
