# Supplementary material for: Quantification of the virus-host interaction in human T lymphotropic virus I infection
Source: Retrovirology. 2005 Dec 9;2:75. doi: 10.1186/1742-4690-2-75 (PMC1327681; doi:10.1186/1742-4690-2-75)
Supplement: Additional File 1 — Description of the general model to describe the relationship between Tax+ and Tax- infected cells in vivo. [file 1742-4690-2-75-S1.pdf]

Additional Material

Modelling the CTL –infected cell interaction in vivo

The general model describing the interaction between infected cells and the CTL response in vivo can be written in the form

$$\frac{dx}{dt} = -(\text{rate of Tax expression})x + (\text{rate of proliferation})x - (\text{rate of death})x + (\text{rate of Tax silencing})y$$
$$\frac{dy}{dt} = +(\text{rate of Tax expression})x + (\text{rate of proliferation})y - (\text{rate of CTL independent death})y$$
$$- (\text{rate of CTL dependent death})y - (\text{rate of Tax silencing})y;$$

where *x* is the proportion of CD4+ lymphocytes that are provirus positive and not expressing Tax and *y* is the proportion of CD4+ lymphocytes that are expressing Tax. This model is represented in diagrammatic form in Figure 3. In this model infected cells that are Tax-negative but positive for other viral proteins are not included. This was to achieve consistency with the CTL lysis assay where CTL lysis was calculated as the proportion of Tax+ cells killed per CD8+ cell per day.

We are interested in the behaviour of provirus positive cells for a given CTL pressure at equilibrium as measured by the CTL lysis assay. For this reason we leave the CTL lysis rate as a variable of the model rather than constructing an explicit equation to describe CTL dynamics. In this model, as in most models of *in vivo* viral dynamics, the exact form of the terms is unknown. For this reason we do not attempt to formulate a model that genuinely describes the virus-CTL interaction. Instead, we simply use a model to suggest mechanisms by which an increase in Tax expression might lead to an increase in proviral load and to make experimentally testable predictions. We made an ad hoc choice of model; the only requirement (which turns out to be surprisingly restrictive) was that it was consistent with previous data [1]. The model used was

$$\dot{x} = -mx - dx + ry$$
$$\dot{y} = 2mx + p - \varepsilon zy - ry - \mu y^2.$$

In this simple model all rates are linear except the rate of proliferation and the rate of CTL-independent death of Tax+ lymphocytes, which are density dependent. The proliferation rate (*p/y*) decreases with increasing activated cell density whereas the death rate (μy) increases. The proportion of HTLV-I-specific CD8+ cells is denoted *z* and they lyse Tax+ cells at a rate ε. Tax- cells start to express Tax on division into two (Tax+) cells. Solving this model for equilibrium we obtain

$$y^* = \frac{\frac{2mr}{m+d} - \varepsilon z - r + \sqrt{\left(\frac{2mr}{m+d} - \varepsilon z - r\right)^2 + 4\mu p}}{2\mu}$$
$$x^* = \frac{r}{m+d} y^*$$

The rate of change of equilibrium proviral load (*x*+y**) with change in Tax expression (*m*) was calculated and is plotted in Figure 4A for a particular random choice of parameters (r=10, m=1, d=1, μ=1, p=1, ε=0…15). We see that *d(x*+y*)/dm* decreases as the CTL clearance rate increases, with a threshold CTL clearance rate beyond which *d(x*+y*)/dm* is negative. Numerically, the rate of change of equilibrium proviral load with net Tax expression (*2mx*-ry**), rather than the rate of Tax expression (*m*), gave qualitatively identical results.

The result that, for low CTL lysis rate, high Tax expression can result in a high proviral load and that the increase in proviral load associated with Tax expression decreases as the strength of the immune response increases [$d(x^*+y^*)/dm>0$, $d(d(x^*+y^*)/dm)/d\varepsilon z<0$] seems intuitively reasonable. So, whilst it is unlikely to be a

property of all plausible models, it is also unlikely to be unique to our particular ad hoc choice of model.

Subjects with High and Low Rates of Tax Expression

Duplicate measurements of Tax expression (proportion of CD4+ cells that were Tax+ after 18h culture) were made for each individual. For every subject except TAC both measurements yielded the same classification into a high of low rate of Tax expression. The individuals who had a low rate of Tax expression (low proportion of CD4+ cells expressing Tax at a given proviral load) were HBF, HS, HY, HBD, TBI, HBH and HAY. Individuals with a high rate of Tax expression load were TW, TAT, TAY, TBA, HT, TAQ, TBG and TAU. Subjects with high and low rates of Tax expression were determined by fitting a least squares regression line through the proviral load- Tax expression data, subjects lying above this line were classed as having a high rate of expression, subjects lying below it were classed as having a low rate of expression (shown in the figure in Additional File 2).

1. Asquith, B., A.J. Mosley, A. Barfield, S.E. Marshall, A. Heaps, P. Goon, E. Hanon, Y. Tanaka, G.P. Taylor, and C.R. Bangham, *A functional CD8+ cell assay reveals individual variation in CD8+ cell antiviral efficacy and explains differences in human T-lymphotropic virus type 1 proviral load.* J Gen Virol. 86(Pt 5): 1515-23. 2005

Additional Figure Legend

Additional Figure: Definition of Subjects with a High and Low Rate of Tax expression

Subjects were classified as having a high or low rate of Tax expression according to their frequency of Tax+ cells (proportion of CD4+ cells expressing Tax protein) at a given proviral load. Since we have controlled for proviral load this amounts to splitting the group into those individuals whose infected CD4+ cells have a high probability or rate of expressing Tax and those with a low probability. This was done by fitting a straight line through the proviral load-Tax expression data using least-squares regression; subjects lying above this line were classed as having a high rate of expression, subjects lying below it were classed as having a low rate of expression.
