# Supplementary figures and images for: Quantification of the virus-host interaction in human T lymphotropic virus I infection
Source: Retrovirology. 2005 Dec 9;2:75. doi: 10.1186/1742-4690-2-75 (PMC1327681; doi:10.1186/1742-4690-2-75)

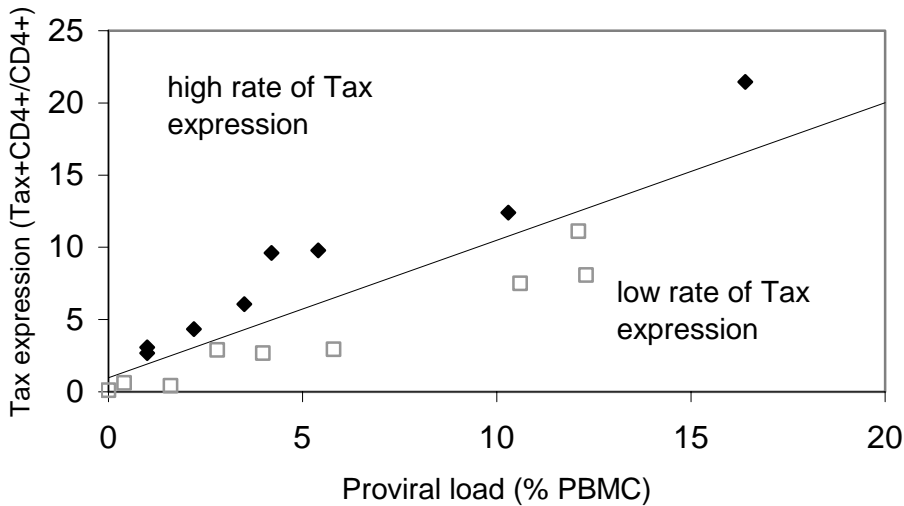

Supplement: Additional File 2 — Figure illustrating the classification of the subject group into individuals whose provirus-positive cells had a high or low rate of Tax expression. [file 1742-4690-2-75-S2.pdf]
